# Rochester Knockings Again

**Published:** 1859-08

**Authors:** 


*Rochester Knockings Again.*—Those of our readers who have been familiar with the Journal since 1851, will doubtless recollect the editorial *exposé* of the famous "Rochester Rappings." We refer to this subject at this time, on account of articles which have appeared in several medical journals, copied from the French, which detail a case of spasmodic contraction of the peroneus brevis, giving rise each time to a loud noise, reported by M. Jobert de Lamballe. This case was successfully treated by subcutaneous section of the tendon. In our July No., Dr. Mack mentions this case in his "Review of Parisian medicine and surgery," saying that M. Robin made a communication on this subject to the *Gazette des Hôpitaux* from the pen of Prof. Flint; but in all other journals where we have seen this subject mentioned, the ingenious investigations of M. Schiff, explaining these mysterious phenomena, have been referred to, while those of Prof. Flint have not been mentioned. In 1854, when Prof. Flint was in Paris, M. Schiff made his communication to the Academy of Sciences; this was nearly three years after the same thing had been published in this country; the *exposé* of the Rochester Rappings having appeared in the Buffalo Medical Journal for March, 1851. In 1854, Prof. Flint made a communication to the Academy of Sciences at Paris, embodying the facts which he had published in 1851, claiming priority over M. Schiff in the discovery that loud noises could be produced by partial luxation and return of the heads of some of the long bones, and by the action of tendons in contraction of certain muscles (Dr. Flint having detailed cases where sounds were produced by the knee-joint, and by the tendons of the peroneus longus and peroneus brevis). His priority was acknowledged by the Academy; and the matter thus rested till the present time.

Prof. Flint made his experiments in connection with Profs. Charles A. Lee and C. B. Coventry, then all of the University of Buffalo. The local excitement in regard to this matter was considerable; an uninterrupted career of two or three years had enabled the Fox and Fish girls (the original spiritual mediums), to make quite a number of proselytes; and the announcement that the secret of the mysterious rappings had been discovered by three learned medical professors, was scouted at by many men of prominence in the city of Buffalo. The operators, however, finally incontinently retired to pursue their occupation in a more favorable field. The rappings in the case of Miss Fox, who was the operating medium, were produced by a partial and voluntary dislocation of the knee-joint. Dr. Flint found another person, a highly respectable lady, who was able to produce the same sounds in the same way. He also found a person who had the power of producing raps by a contraction of the peroneus longus, causing the tendon to slip out of its groove with a loud noise, and others who did the same with the peroneus brevis.

These facts are undoubtedly all familiar to the old readers of our Journal; and we are a little surprised that the journals in which have appeared the articles which we mentioned, giving the credit of priority to M. Schiff, should have ignored the claims of Prof. Flint.

